# Retraction: First-principle investigations of structural, electronic, magnetic and optical properties of bulk BiVO_3_

**DOI:** 10.1039/c9ra90029b

**Published:** 2019-04-26

**Authors:** Andrew Shore

**Affiliations:** Royal Society of Chemistry Thomas Graham House, Science Park, Milton Road Cambridge UK advances-rsc@rsc.org

## Abstract

Retraction of ‘First-principle investigations of structural, electronic, magnetic and optical properties of bulk BiVO_3_’ by Biao Liu *et al.*, *RSC Adv.*, 2016, **6**, 92473–92478.

The Royal Society of Chemistry hereby wholly retracts this *RSC Advances* article as recent recalculations on the data have shown that the results in the paper are unreliable.

The original calculation of the DOS (density of states) of BiVO_3_ in the C-AFM structure is inaccurate. The authors have also recalculated the energies of the FM, A-AFM, C-AFM and G-AFM structures and found that, although the C-AFM phase is the lowest energy structure, the energy difference values of the FM, A-AFM and G-AFM relative to C-AFM are inaccurate in the paper. In addition, the authors found that the structural data for BiVO_3_ with *Pmna* and *C*2/*C* structures in Table 1 is incorrect.

The recalculated results mean that the conclusions of the published paper are now unreliable; therefore, the article is being retracted to protect the accuracy and integrity of the scientific record.

The corresponding author, Meng-Qiu Cai, agreed to retract the article, but did not respond to any correspondence regarding the wording of the retraction notice. The co-authors, Biao Liu, Li-Juan Wu, Yu-Qing Zhao and Lin-Zhi Wang, were contacted but did not respond.

Signed: Andrew Shore, Executive Editor, *RSC Advances*

Date: 10^th^ April 2019

## Supplementary Material

